# PEtOx-DOPE nanoliposomes functionalized with peptide 563 in targeted *BikDDA* delivery to prostate cancer

**DOI:** 10.55730/1300-0152.2693

**Published:** 2024-05-23

**Authors:** Ayca Ece NEZİR, Zeynep Büşra BOLAT, Ongun Mehmet SAKA, Itır Ebru ZEMHERİ, Sevgi GÜLYÜZ, Umut Uğur ÖZKÖSE, Özgür YILMAZ, Asuman BOZKIR, Fikrettin ŞAHİN, Dilek TELCİ

**Affiliations:** 1 Department of Genetics and Bioengineering, Faculty of Engineering, Yeditepe University, İstanbul, Turkiye; 2 Department of Molecular Biology and Genetics, Hamidiye Institute of Health Sciences, University of Health Sciences, İstanbul, Turkiye; 3 Experimental Medicine Research and Application Center, University of Health Sciences, İstanbul, Turkiye; 4 Department of Pharmaceutical Technology, Faculty of Pharmacy, Ankara University, Ankara, Turkiye; 5 Department of Pathology, Ümraniye Training and Research Hospital, University of Health Sciences, İstanbul, Turkiye; 6 Materials Technologies, Marmara Research Center, TÜBİTAK, Kocaeli, Turkiye; 7 Department of Chemistry, Faculty of Science and Letters, İstanbul Technical University, İstanbul, Turkiye; 8 Department of Chemistry, Faculty of Science and Letters, Piri Reis University, İstanbul, Turkiye

**Keywords:** Prostate cancer, poly(2-ethyl-2-oxazoline), liposome, *BikDDA*, peptide 563, targeted gene therapy

## Abstract

**Background:**

Nanocarrier-based systems have cultivated significant improvements in prostate cancer therapy. However, the efforts are still limited in clinical applicability, and more research is required for the development of effective strategies. Here, we describe a novel nanoliposomal system for targeted apoptotic gene delivery to prostate cancer.

**Methods:**

Poly (2-ethyl-2-oxazoline) (PEtOx) dioleoyl phosphatidylethanolamine (DOPE) nanoliposomes were conjugated with the prostate-specific membrane antigen (PSMA)-targeting peptide GRFLTGGTGRLLRIS (P563) and loaded with *BikDDA*, a mutant form of the proapoptotic Bik. We selected 22Rv1 cells with moderate upregulation of PSMA to test the in vitro uptake, cell death, and in vivo anticancer activity of our formulation, P563-PEtOx-DOPE-BikDDA.

**Results:**

*BikDDA* was upregulated in 22Rv1 cells, inducing cell death, and CD-1 nude mice xenografts administered with the formulation showed significant tumor regression.

**Conclusion:**

We suggest that P563-PEtOx-DOPE-BikDDA nanoliposomes can serve as prominent gene carriers against prostate cancer.

## 1. Introduction

Prostate cancer is one of the most frequently diagnosed malignancies among men worldwide (Siegel et al., 2022). Despite the notable advance in the experimental therapy of prostate cancer enabled by nanomaterial-based systems, in vivo precision-targeting, biocompatibility, and safety issues continue to pose major limitations in actual clinical application (Pranav et al., 2023). Therefore, profound research is required for developing safe and efficient nanodelivery systems for prostate cancer therapy.

While nanoparticles (NPs) can reach tumors through the enhanced permeability and retention (EPR) effect, achieving passive targeting due to the leaky tumor vasculature, active targeting is commonly utilized to further advance in vivo specificity (Batool et al., 2023). Prostate-specific membrane antigen (PSMA) is a prominent target given that it is upregulated on prostate cancer cell surfaces as well as the neovasculature of all major solid tumors (Chang et al., 1999). Our previous work showed that prostate cancer cells with moderate to high levels of PSMA expression could be effectively targeted by peptide 563 (P563; GRFLTGGTGRLLRIS), a highly selective PSMA-targeting peptide (Shen et al., 2013). Building on these findings, our group synthesized P563-conjugated PEtOx-DOPE (dioleoyl phosphatidylethanolamine) lipopolymers for targeted gene delivery to prostate cancer (Gulyuz et al., 2021). As for the cargo molecule, we selected *BikDDA*, a mutant form of the proapoptotic gene Bik, which has a prolonged half-life and possesses increased apoptotic activity (Jiao et al., 2014).

Here, we tested the novel P563-PEtOx-DOPE-BikDDA nanoliposome formulation for antitumor activity against prostate cancer in vitro, using 22Rv1 cells exhibiting PSMA expression, and in vivo, on 22Rv1 xenograft models. To the best of our knowledge, this is the first study that employs the use of the *BikDDA* gene as a treatment strategy for prostate cancer.

## 2. Materials and methods

### 2.1. Site-directed mutagenesis

The *BikDDA* cargo gene used in this study was obtained previously, as described in Oz et al. (2020). Briefly, the QuikChange Lightning Site-Directed Mutagenesis Kit was used to modify the Bik gene: Thr33 and Ser35 residues were mutated to aspartic acid, and the Ser124 residue was replaced with an alanine to obtain *BikDDA*.

### 2.2. Cell culture

PNT1A human prostate epithelial cells (95012614; Sigma-Aldrich, USA) and 22Rv1 prostate carcinoma cells (CRL-2505; American Type Culture Collection [ATCC]) were routinely cultured in Roswell Park Memorial Institute (RPMI) 1640 medium (Gibco, Thermo Fisher Scientific, USA) supplemented with 10% (v/v) fetal bovine serum (FBS; Gibco) and 100 units/mL penicillin/streptomycin (Gibco). The cells were maintained in a humidified incubator at 37 °C and 5% CO_2_.

### 2.3. Quantitative real-time PCR

Total RNA was isolated from 22Rv1 cells (3 × 10^5^ cells/well in 6-well plates) using a TRIzol reagent (Invitrogen), and cDNA was synthesized using Sensiscript reverse transcription kit (QIAGEN), following the respective instructions. Both 18S rRNA (QuantiTect Primer Assay, QIAGEN, Germany) and Bik forward (5′-GAGACATCTTGATGGAGACC-3′) and reverse (5′-TCTAAGAACATCCCTGATGT-3′) primers were used to amplify the respective PCR products (Bio-Rad, CFX96 Real-Time PCR System). The data was analyzed using CFX Manager.

### 2.4. Cell viability

P563-PEtOx-DOPE polymers were successfully synthesized (Gulyuz et al., 2021), and P563-PEtOx-DOPE nanoliposomes were prepared using the lipid hydration method, as previously demonstrated (Saka and Bozkır, 2018).

The effect of P563-PEtOx-DOPE-BikDDA nanoliposomes on 22Rv1 prostate cancer cell viability was assessed using a WST-1 cell proliferation reagent (Roche, USA). The 22Rv1 cells seeded into 96-well plates at a density of 5 × 10^4^ cells/well were treated with 10 μg/mL of BikDDA plasmid (naked *BikDDA*) or nanoliposomes carrying an equal concentration of the gene P563-PEtOx-DOPE-BikDDA. The cells were also treated with an equivalent dose of the empty nanoliposome formulation (P563-PEtOx-DOPE). Following 72 h of treatment, WST-1 reagent was added at a dilution of 1:10 (v/v) in RPMI. Absorbance was measured at 450 nm using SpectraMax Paradigm Multimode Microplate Reader (Molecular Devices, San Jose, CA, USA). Untreated (control) cells were considered 100% viable, and the relative viability of cells under different treatment conditions was compared accordingly.

### 2.5. Cell death assay

At a density of 3 × 10^4^ cells/well, 22Rv1 cells were seeded into 8-well chambers. Subsequently, they underwent treatment with P563-PEtOx-DOPE-BikDDA, P563-PEtOx-DOPE, or 1 μM staurosporine (positive control). The cells were fixed with paraformaldehyde (4% w/v) and permeabilized with a Triton X-100 (0.1% v/v) solution in sodium citrate (0.1% w/v). In between each step, the cells were washed three times with PBS. In Situ Cell Death Detection Kit (Roche, USA) was used to analyze cell death, following the manufacturer’s protocol: the TUNEL reaction mixture was prepared, and the samples were labeled at 37 °C for 75 min. The cells were visualized using a Zeiss Axioscope fluorescent microscope, and average fluorescent intensity was calculated from five independent images for each condition using ImageJ.

### 2.6. Serum stability

P563-PEtOx-DOPE-BikDDA nanoliposomes in PBS were stored at 4 °C in amber tubes for two weeks. Nanoliposomes were mixed with Dulbecco’s modified Eagle’s medium (DMEM) containing 10% FBS at a 1:50 (v/v) ratio and incubated at 37 °C for 24 h. Serum stability was assessed by the size distribution and zeta potential of the nanoliposomes (Chan et al., 2009; Jones and Nicholas, 1991). The pH was approximately 7.4 for all media utilized during the characterization tests, including water, buffers, and serum-containing DMEM.

### 2.7. Assessment of in vivo antitumor activity

Athymic male CD-1 nu/nu mice were obtained from Charles River Laboratories (Germany) and allowed to rest in the animal facility of Yeditepe University (Türkiye) for two weeks before tumor inoculation. The animals were maintained under a 12 h light/12 h dark cycle and had ad libitum access to food and water. On the day of inoculation, the mice were injected with 22Rv1 cells in a Matrigel/PBS mixture as described in our previous work (Nezir et al., 2023). The mice were randomly assigned into P563-PEtOx-DOPE-BikDDA, P563-PEtOx-DOPE, naked *BikDDA*, and control (PBS) groups on the first day of injection and received a total of 8 injections over the course of 28 days. *BikDDA* administered in the naked or encapsulated form was 7 μg/injection/mice, while P563-PEtOx-DOPE was administered at a dose equivalent to the administered *BikDDA*-loaded nanoliposomes. Tumor tissues were collected following cervical dislocation, and tumor weight and volume data were collected. All tissues were stored in a 10% formalin solution until pathological analyses. The following formula was used to calculate tumor volumes: tumor volume (mm^3^) = 1/2 × length × width^2^—length, long radius; width, short radius (Zhao et al., 2022). The study protocol was approved by the Animal Care and Welfare Committee of Yeditepe University (Türkiye, decision number: 644), and all experiments were performed in accordance with the ARRIVE guidelines.

### 2.8. Histopathological evaluation

All samples were transferred to Ümraniye Training and Research Hospital, Department of Pathology, for histopathological evaluations. Paraffin-embedded tissue sections were prepared from formalin-fixed tumor tissues and subjected to hematoxylin and eosin (H&E) staining as described previously (Gulyuz et al., 2021; Nezir et al., 2023). A score of 0 to 3 (0, absent; 1, mild; 2, moderate; 3, severe) was assigned to each sample in terms of necrosis, inflammation, and fibrosis.

### 2.9. Statistical analysis

Differences between group means were analyzed in GraphPad Prism 8 (GraphPad Software, USA) using one-way analysis of variance followed by Tukey posthoc test, and the data are expressed as mean ± standard deviation (SD). A two-sided p value of <0.05 was considered statistically significant.

## 3. Results

### 3.1. Serum stability of P563-PEtOx-DOPE-BikDDA

The particle size and zeta potential values of P563-PEtOx-DOPE-BikDDA are shown in Table 1. A 30-nm difference was observed in particle size after incubation of the nanoliposomes in serum, indicating a slight interaction between the nanoliposomes and serum proteins. While the zeta potential of the formulation increased significantly following serum incubation, no significant change was observed in charge distribution in the medium. No aggregation of the liposomes was observed at any phase of characterization, indicating the suitability of the materials to be tested under in vivo conditions.

### 3.2. BikDDA transfection efficiency of P563-PEtOx-*DOPE* nanoliposomes

**Figure 1:**
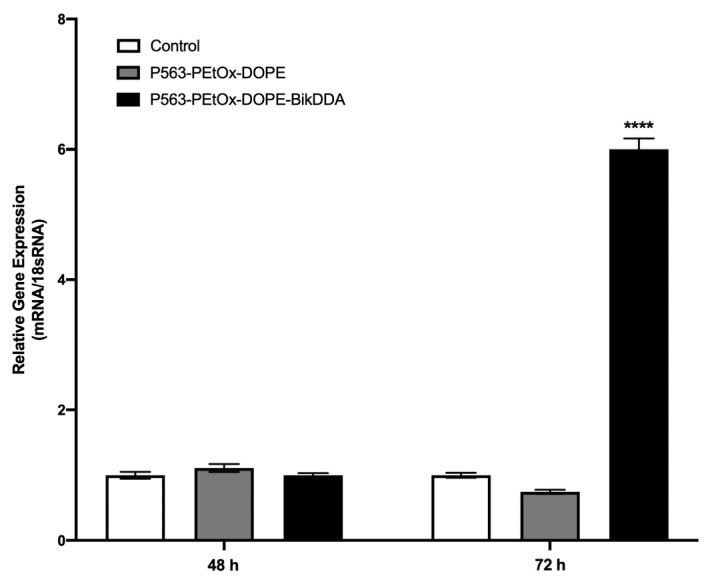
Relative *BikDDA* expression in 22Rv1 cell lines transfected with P563-PEtOx-DOPE-BikDDA nanoliposomes. The results were compared with those of untreated (control) and empty nanoliposome (P563-PEtOx-DOPE)-treated cells. The reference gene used was 18s rRNA. ^****^ p ≤ 0.001.

*BikDDA* mRNA expression level in 22Rv1 prostate cancer cells transfected with P563-PEtOx-DOPE-BikDDA nanoliposomes was increased by 6-fold compared with untreated (control) cells and those treated with empty nanoliposomes (P563-PEtOx-DOPE) after 72 h of treatment, while no change in mRNA levels was observed at 48 h (Figure 1). These results imply that P563-PEtOx-DOPE-BikDDA could effectively increase the expression of *BikDDA* in 22Rv1 cells, given an appropriate treatment duration.

### 3.3. The effect of P563-PEtOx-DOPE-BikDDA on cell viability

**Figure 2:**
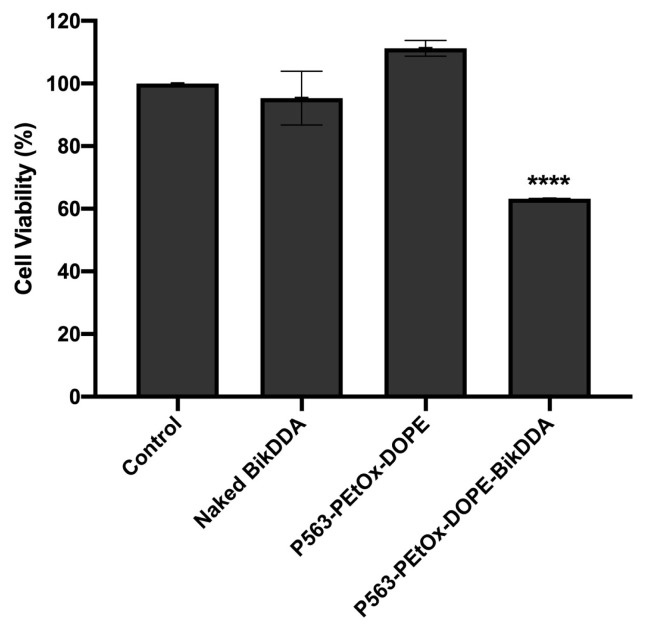
The effect of P563-PEtOx-DOPE-BikDDA on 22Rv1 cell viability. The cells were treated with P563-PEtOx-DOPE-BikDDA, an equal dose of naked *BikDDA*, or an equal dose of the empty nanoliposomes (P563-PEtOx-DOPE). Percentage cell viability was assessed by assuming the viability of untreated (control) cells to be 100%. ^****^ p ≤ 0.001.

A significant decrease to 60% viability was observed when 22Rv1 cells were treated with P563-PEtOx-DOPE-BikDDA for 72 h (Figure 2). An equal dose of naked *BikDDA* or the empty carrier (P563-PEtOx-DOPE) did not promote a significant change in cell viability compared with untreated (control) cells.

### 3.4. The effect of P563-PEtOx-DOPE-BikDDA on cell death

**Figure 3:**
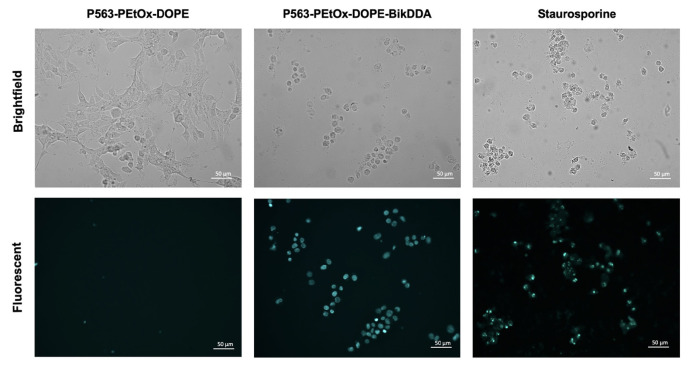
Representative fluorescent microscopy images showing 22Rv1 cell death detected by the TUNEL assay. DNA double-strand breaks were observed upon treatment with P563-PEtOx-DOPE-BikDDA and staurosporine (positive control). Objective: 20×; scale bars: 50 μm.

A TUNEL assay was performed to analyze cell death. Average fluorescent intensities calculated from five independent areas revealed a 6.2-fold increased signal intensity for P563-PEtOx-DOPE-BikDDA and 4.3-fold for staurosporine (positive control), compared with P563-PEtOx-DOPE (empty nanoliposomes). The change in cell morphology induced by the treatment conditions and the corresponding fluorescent images are shown in Figure 3.

### 3.5. In vivo antitumor efficacy of P563-PEtOx-DOPE-BikDDA

**Figure 4:**
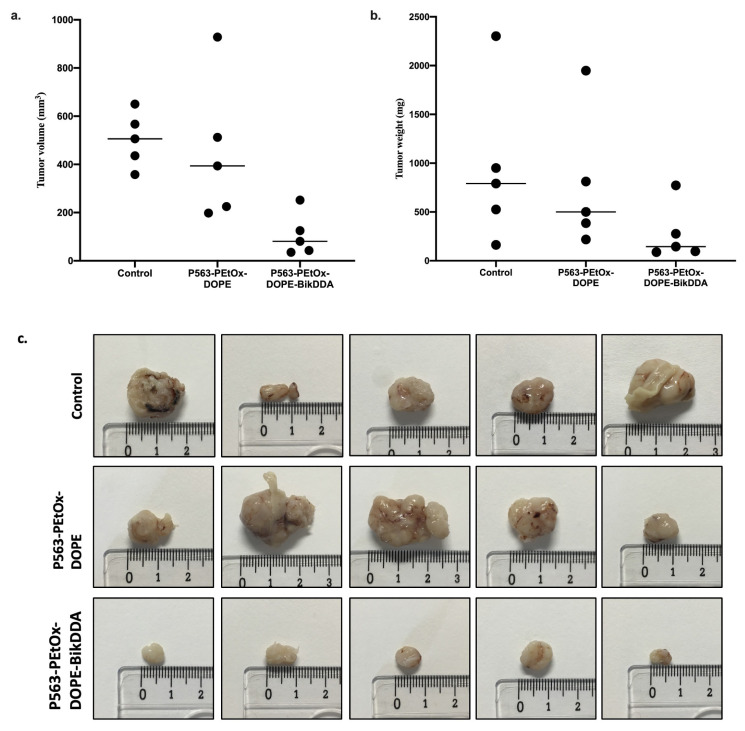
Changes in (a) tumor volume and (b) weight of CD-1 nu/nu male 22Rv1 tumor xenografts (n = 5) upon treatment with the vehicle control (PBS), P563-PEtOx-DOPE, or P563-PEtOx-DOPE-BikDDA. (c) Images of tumors in all groups (n = 5).

Mice administered with P563-PEtOx-DOPE-BikDDA had a significantly lower average tumor volume compared with both the control group that received PBS (107.2 vs. 503.4 mm^3^, p < 0.5) and P563-PEtOx-DOPE (107.2 vs. 451.5 mm^3^, p < 0.5) (Figure 4a). Average tumor weight in P563-PEtOx-DOPE-BikDDA, P563-PEtOx-DOPE, and control groups were 275.8, 772.8, and 946.4 mg, respectively, confirming the significant reduction in tumors in the P563-PEtOx-DOPE-BikDDA group (p < 0.5; Figure 4b). Tumors collected from all animals are shown in Figure 4c. These results indicated that *BikDDA* transfected in our nanoliposome formulation could effectively promote tumor regression in vivo.

All tumor samples were histopathologically confirmed to have a Gleason score (GS) of 5 + 5, indicating the successful establishment of the xenograft models. The average necrosis score was 1.3, inflammation was 1.0, and fibrosis was 1.0 in the control group mice that received PBS injections (Table 2). Similarly, these scores were 1.2, 0.8, and 1.0, respectively, in the P563-PEtOx-DOPE (empty nanoliposome) group. The lowest scores were assigned to the P563-PEtOx-DOPE-BikDDA group, with a score of 0.5 for all three factors (Table 2).

## 4. Discussion

Nanocarrier-based cancer therapies have yielded promising results in recent years. A rather successful example is BIND-014 (Hrkach et al., 2012), a polymeric nanoparticle (NP) formulation comprising poly(D,L-lactide) (PLA) and poly(ethylene glycol) (PEG), proven to be safe for human use (Avgoustakis, 2004). The NP encapsulated docetaxel, a chemotherapeutic drug efficient against prostate cancer, and was functionalized with a small molecule ligand targeting the extracellular domain of prostate-specific membrane antigen (PSMA) that is upregulated on prostate cancer cell surfaces as well as the neovasculature of all major solid tumors (Von Hoff et al., 2016). The formulation showed prolonged circulation in the vascular system, suppressed tumor growth in more than one animal model, and enabled tumor regression in patients at a dose lower than that used in clinical practice for the soluble form of docetaxel (Hrkach et al., 2012; Von Hoff et al., 2016). Most recently, in a phase 2 study, the number of PSMA-positive circulating tumor cells was reduced in patients treated with BIND-014. However, the study lacked comparison with soluble docetaxel, and a high ratio of the patients experienced adverse effects, including neuropathy (Autio et al., 2018).

Yet, only a few of the numerous nanodelivery systems developed to this day are in clinical trials. Therefore, extensive research has been focused on developing novel nanocarrier-based delivery systems. Recently, we developed a polymeric micelle formulation similar to BIND-014 in that it was loaded with a low dose of docetaxel and achieved active targeting through the tumor-homing peptide P563, a peptide with high affinity to PSMA (Nezir et al., 2023). Our formulation induced tumor regression in 22Rv1 xenografts and displayed a good safety profile.

In the present study, we tested a nanoliposome formulation employing the same PSMA-targeting approach for in vivo targeting of prostate cancer using 22Rv1 xenografts. This setting was designed to evaluate the potential of our formulation in targeted gene therapy. Therefore, instead of docetaxel, the cargo molecule was the apoptotic *BikDDA* gene. During apoptosis, p53 induces the transcription of several genes that produce BCL-2 homology domain 3-only proteins, including the proapoptotic Bik gene (Hao et al., 2023). The action of the protein product Bik, which is localized in the endoplasmic reticulum, mobilizes calcium ions to the mitochondria, resulting in the remodeling of the mitochondrial cristae and the induction of apoptosis through the mitochondrial pathway (Chinnadurai et al., 2008). Previously, *BikDDA* was shown to enhance apoptotic activity in triple-negative breast cancer cells, compared to another mutant, *BikDD* (Jiao et al., 2014). Here, for the first time in the literature, we have tested the apoptotic effect of the *BikDDA* gene in prostate cancer by targeting the androgen responsive 22Rv1 cell line and their in vivo xenograft model. In vitro assays showed that 22rv1 cells subjected to P563-PEtOx-DOPE-BikDDA were efficiently transfected with the gene, and apoptotic cell death was effectively induced by this longer half-life mutant form of Bik. In vivo, tumor volume and tumor weight were significantly reduced in mice administered with P563-PEtOx-DOPE-BikDDA. Moreover, tumor necrosis was reduced in the treatment group compared with the PBS and empty carrier controls, indicating a less aggressive tumor profile (Liu and Jiao, 2020). Overall, these results suggest that P563-PEtOx-DOPE nanoliposomes can serve as a promising gene delivery system to induce tumor regression.

## 5. Conclusion

In summary, this study highlights several important findings in prostate cancer therapy: (i) delivery of *BikDDA*, a mutated form of the proapoptotic Bik, can be considered a promising strategy in the field of gene therapy; (i) PEtOx-DOPE nanoliposomes can serve as safe and effective gene delivery platforms; and (iii) targeting of prostate cancer cells in vitro and in vivo can be achieved by employing the PSMA-targeting peptide P563, even for cells with only moderate upregulation of PSMA on the cell surface. Therefore, the findings presented here are expected to have a guiding significance for future advances in nanocarrier-based prostate cancer therapy.

## Figures and Tables

**Table 1:** Particle size and zeta potential values of P563-PEtOx-DOPE-BikDDA.

	Particle size (nm)		Zeta potential (mV)	
	Before incubation	After incubation	Before incubation	After incubation
**P563-PEtOx-DOPE-BikDDA**	146.0 ± 1.557	117.3 ± 3.936	−30.9 ± 0.794	−11.8 ± 0.153
**DMEM with 10% FBS**	-	148.0 ± 0.2082	-	−13.6 ± 1.0000

The values represent the mean ± SD of three independent experiments.

**Table 2:** Pathological evaluation of the tumors in all groups.

Group (n = 5)	Necrosis	Inflammation	Fibrosis
Control	1.3	1.0	1.0
P563-PEtOx-DOPE	1.2	0.8	1.0
P563-PEtOx-DOPE-BikDDA	0.5	0.5	0.5

The values represent the average of five pathological scores assigned to each group.

## References

[b1-tjb-48-03-174] Autio KA Dreicer R Anderson J Garcia JA Alva A 2018 Safety and efficacy of BIND-014, a docetaxel nanoparticle targeting prostate-specific membrane antigen for patients with metastatic castration-resistant prostate cancer: a phase 2 clinical trial JAMA Oncology 4 10 1344 1351 10.1001/jamaoncol.2018.2168 29978216 PMC6233779

[b2-tjb-48-03-174] Avgoustakis K 2004 Pegylated poly(lactide) and poly(lactide-co-glycolide) nanoparticles: preparation, properties and possible applications in drug delivery Current Drug Delivery 1 4 321 333 10.2174/1567201043334605 16305394

[b3-tjb-48-03-174] Batool S Sohail S Ud Din F Alamri AH Alqahtani AS 2023 A detailed insight of the tumor targeting using nanocarrier drug delivery system Drug Delivery 30 1 2183815 https://doi.org/10.1080%2F10717544.2023.2183815 36866455 10.1080/10717544.2023.2183815 PMC10191068

[b4-tjb-48-03-174] Chan JM Zhang L Yuet KP Liao G Rhee JW 2009 PLGA-lecithin-PEG core-shell nanoparticles for controlled drug delivery Biomaterials 30 8 1627 1634 10.1016/j.biomaterials.2008.12.013 19111339

[b5-tjb-48-03-174] Chang SS O’Keefe DS Bacich DJ Reuter VE Heston WDW 1999 Prostate-specific membrane antigen is produced in tumor-associated neovasculature Clinical Cancer Research 5 10 2674 2681 10537328

[b6-tjb-48-03-174] Chinnadurai G Vijayalingam S Rashmi R 2008 BIK, the founding member of the BH3-only family proteins: mechanisms of cell death and role in cancer and pathogenic processes Oncogene 27 Suppl 1 S20 S29 10.1038/onc.2009.40 19641504 PMC2928562

[b7-tjb-48-03-174] Gulyuz S Bayram D Ozkose UU Bolat ZB Kocak P 2021 Synthesis, biocompatibility and gene encapsulation of poly(2-ethyl 2-oxazoline)-dioleoyl phosphatidylethanolamine (PEtOx-DOPE) and post-modifications with peptides and fluorescent dye coumarin International Journal of Polymeric Materials and Polymeric Biomaterials 70 14 981 993 10.1080/00914037.2020.1767617

[b8-tjb-48-03-174] Hao Q Chen J Lu H Zhou X 2023 The ARTS of p53-dependent mitochondrial apoptosis Journal of Molecular Cell Biology 14 10 mjac074 10.1093/jmcb/mjac074 36565718 PMC10053023

[b9-tjb-48-03-174] Hrkach J Von Hoff D Mukkaram Ali M Andrianova E Auer J 2012 Preclinical development and clinical translation of a PSMA-targeted docetaxel nanoparticle with a differentiated pharmacological profile Science Translational Medicine 4 128 128ra39 10.1126/scitranslmed.3003651 22491949

[b10-tjb-48-03-174] Jiao S Wu M Ye F Tang H Xie X 2014 BikDDA, a mutant of Bik with longer half-life expression protein, can be a novel therapeutic gene for triple-negative breast cancer PLoS ONE 9 3 e92172 10.1371/journal.pone.0092172 24637719 PMC3956915

[b11-tjb-48-03-174] Jones MN Nicholas AR 1991 The effect of blood serum on the size and stability of phospholipid liposomes Biochimica et Biophysica Acta (BBA) - Biomembranes 1065 2 145 152 10.1016/0005-2736(91)90224-v 2059649

[b12-tjb-48-03-174] Liu ZG Jiao D Necroptosis, tumor necrosis and tumorigenesis (2020) Cell Stress 4 1 1 8 https://doi.org/10.15698%2Fcst2020.01.208 10.15698/cst2020.01.208 PMC6946014 31922095

[b13-tjb-48-03-174] Nezir AE Bolat ZB Ozturk N Kocak P Zemheri E 2023 Targeting prostate cancer with docetaxel-loaded peptide 563-conjugated PEtOx-co-PEI_30%_-*b*-PCL polymeric micelle nanocarriers Amino Acids 55 8 1023 1037 10.1007/s00726-023-03292-3 37318626

[b14-tjb-48-03-174] Oz UC Bolat ZB Poma A Guan L Telci D 2020 Prostate cancer cell-specifc BikDDA delivery by targeted polymersomes Applied Nanoscience 10 3389 3401 10.1007/s13204-020-01287-0

[b15-tjb-48-03-174] Pranav Laskar P Jaggi M Chauhan SC Yallapu MM 2023 Biomolecule-functionalized nanoformulations for prostate cancer theranostics Journal of Advanced Research 51 197 217 10.1016/j.jare.2022.11.001 36368516 PMC10491979

[b16-tjb-48-03-174] Saka OM Bozkır A 2018 Preparation and evaluation of Tsp-1 loaded pegylated cationic liposomes for inhibiting angiogenesis International Journal of Biotechnology and Bioengineering 4 1 01 06

[b17-tjb-48-03-174] Shen D Xie F Edwards WB 2013 Evaluation of phage display discovered peptides as ligands for prostate-specific membrane antigen (PSMA) PLoS ONE 8 7 1 8 10.1371/journal.pone.0068339 PMC3723849 23935860

[b18-tjb-48-03-174] Siegel RL Miller KD Fuchs HE Jemal A 2022 Cancer statistics, 2022 CA: A Cancer Journal for Clinicians 72 1 7 33 10.3322/caac.21708 35020204

[b19-tjb-48-03-174] Von Hoff DD Mita MM Ramanathan RK Weiss GJ Mita AC 2016 Phase I study of PSMA-targeted docetaxel-containing nanoparticle BIND-014 in patients with advanced solid tumors Clinical Cancer Research 22 13 3157 3163 10.1158/1078-0432.ccr-15-2548 26847057

[b20-tjb-48-03-174] Zhao Y Dandan S Shang M Sun X Guo L 2022 GRP78-targeted and doxorubicin-loaded nanodroplets combined with ultrasound: a potential novel theranostics for castration-resistant prostate cancer Drug Delivery 29 203 213 10.1080/10717544.2021.2023698 34985396 PMC8741251

